# Health Impacts of High BMI in China: Terrible Present and Future

**DOI:** 10.3390/ijerph192316173

**Published:** 2022-12-02

**Authors:** Hong Xiang, Runjuan Yang, Jiaxin Tu, Xi Guan, Xufeng Tao

**Affiliations:** 1 Laboratory of Integrative Medicine, First Affiliated Hospital of Dalian Medical University, Dalian 116011, China; 2 Department of Pharmacy, First Affiliated Hospital of Dalian Medical University, Dalian 116011, China

**Keywords:** high body mass index, China, health effects, temporal trend, projection, global burden of disease study

## Abstract

Temporal trends and future expectations of health effects due to high body mass index (BMI) remain uncertain in China. The trends of high-BMI-related death in China were evaluated and predicted until 2040 using data and methods from the Global Burden of Disease study. The absolute numbers and age-standardized rates of death and disability-adjusted life years (DALYs) were also calculated by age, gender, and cause. From 1990 to 2019, the high-BMI-related death percent, number and rate were all significantly increased in China, and death rate may exceed that globally in the next 10 years. High BMI caused more deaths and DALYs for men than for women, and the gap appeared to increase over time. In 2019, the burden of high BMI among ages 0–14 and 15–19 for children and adolescents were lower than that among adults (>20 years). The most common cause of death associated with high BMI was stroke, followed by ischemic heart disease and hypertensive heart disease. High BMI burden is a significant public health challenges in China. BMI surveillance and evaluation of evidence-based preventive strategies should be immediately initiated in Chinese residents due to the rapid increase in the burden of high BMI.

## 1. Introduction

Globally, overweight and obesity are on the rise, currently affecting approximately 30% of the world population, and have been associated with a range of health, psycho-behavioral and social issues [1,2,3]. The concept of body mass index (BMI), first proposed by Quételet, is the most commonly used standard to determine the degree of overweight and obesity. The BMI is calculated as the ratio of the weight (weight in kilograms) to the height squared [4]. Studies have identified high BMI as an independent risk factor for chronic diseases, such as cardiovascular disease [5], diabetes mellitus [6], chronic kidney disease [7], nonalcoholic fatty liver disease (NAFLD) [8], cancers [9], and musculoskeletal disorders [10]. On the other hand, high BMI may be characterized as a predictive factor for poor social function of individuals, who experience frequent weight-related discrimination and appearance-based social anxiety. Most individuals with high BMI are prone to low self-esteem, timidity and even depression, social barriers, etc., mainly manifesting in the form of rebellious character, lack of communication with people around them, or subconsciously avoiding the negative evaluation of obesity [11,12].

In recent years, as global health organizations work to develop prevention policies to combat overweight and obesity, within and across nations, increasing efforts have been directed towards quantifying the potential effects of high BMI on a variety of health outcomes [13,14,15]. However, China rarely discloses information about the health effects of high BMI in a timely fashion. Thus, the public and health policymakers allocate little attention to improving high BMI, prioritizing other clinical fields instead. To fill this knowledge gap, we systematically evaluated the trends in the pattern of deaths and disability-adjusted life-years (DALYs) in China related to high BMI based on age and gender, 1990–2019. Public health policymakers can confidently develop and implement targeted programs aimed at preventing and managing overweight and obesity by having comprehensive and up-to-date knowledge of the overall burden of high BMI.

## 2. Materials and Methods

### 2.1. Data Sources

The Global Burden of Disease (GBD) Study 2019, coordinated by the Institute for Health Metrics and Evaluation (IHME) covering more than 50,000 data sources worldwide, evaluated the health effects of high BMI. The GBD Results Tool (https://vizhub.healthdata.org/gbd-results/ (accessed on 24 August 2022)) analyzed 369 diseases and injuries, and 87 risk factors from over 204 countries and territories, during the period 1990–2019. GBD Foresight (https://vizhub.healthdata.org/gbd-foresight/ (accessed on 24 August 2022)) provides an open access visualization tool for predicting risk factors and disease mortality by age and sex across countries until 2040. The detailed methodologies of GBD 2019 and the comparative risk assessment for high BMI have already been described elsewhere [13,16]. 

### 2.2. Statistical Analysis

An adult with a high BMI is defined as one who has a BMI ≥ 25 kg/m^2^ according to the International Obesity Task Force’s guidelines for ages < 20 years [13]. Using the GBD data and methods, we systematically compared the percent, number and rate of death between China and the rest of the world from 1990 to 2019, and predicted death trends until 2040. We also estimated the sex- and age-specific burden related to high BMI in China between 1990 and 2019 [16,17]. Moreover, we artificially divided the Chinese population into 3 age groups: ages 0–14 for children, ages 15–19 for adolescents, and ages > 20 years for adults, and summed their data as the new age group. The health burden was quantified by deaths and DALYs, which were reported as numbers and age-standardized rates (ASRs). DALYs is a composite metric based on the sum of years lived with disability and years lost due to high BMI. Data were presented as values with a 95% uncertainty interval (UI). In addition, the estimated annual percentage change (EAPC) from 1990 to 2019 was also calculated to reflect the change trends in age-standardized death rates (ASDRs) and ASR-DALYs based on the formula 100 × (exp (β) − 1) (Y = α + βX + ε, Y = ln (ASR), X = calendar year, ε = the error term), and a linear regression model was used to calculate the 95% confidence interval (CI). All data were analyzed using R package 4.2.0 or GraphPad Prism 6.0.

## 3. Results

### 3.1. The Mortality of High BMI in China May Exceed That in Global in the Next 10 Years

**Figure 1:**
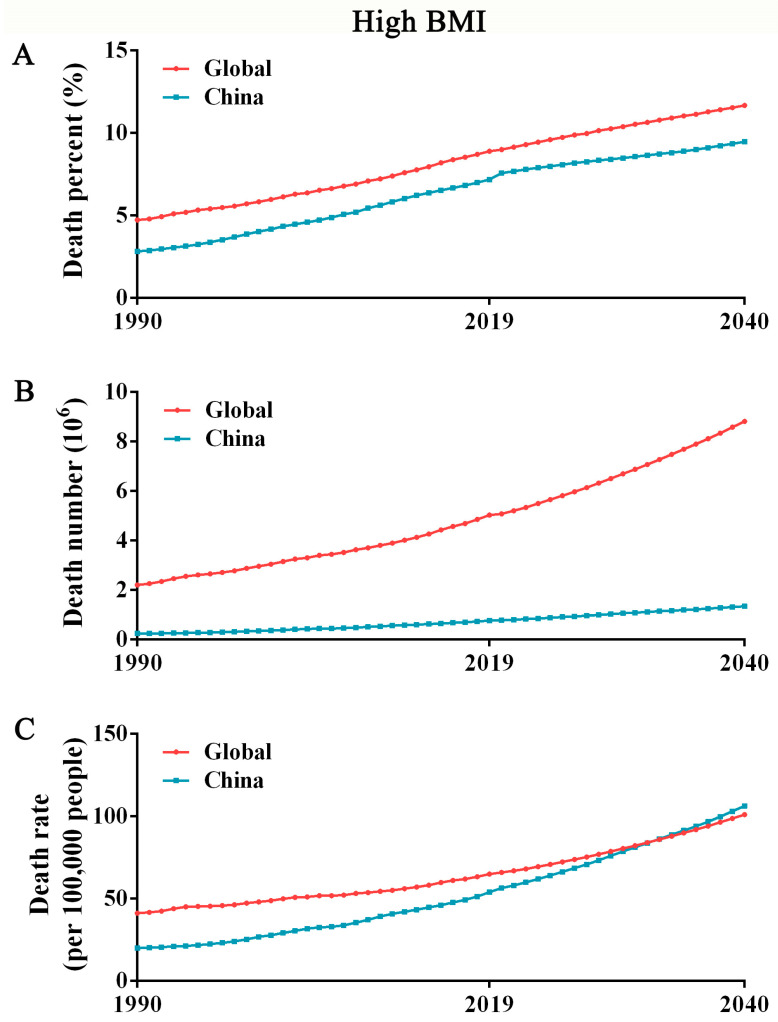
Death trends of high BMI globally and in China from 1990 to 2040. (**A**) Death percentage attributed to high BMI globally and in China from 1990 to 2040. (**B**) Death numbers attributed to high BMI globally and in China from 1990 to 2040. (**C**) Death rate attributed to high BMI globally and in China from 1990 to 2040.

The overall burden of high BMI is primarily attributed to the change in the number of deaths associated with high BMI; thus, our analyses focused on the global and Chinese dynamics of the high-BMI-related death burden from 1990 to 2019. The total death burden due to high BMI is presented as percentage, number and rate. Both globally and in China, the high-BMI-related total death percentage, number and rate all significantly increased between 1990 and 2019. In China, the percentage of deaths increased from 2.82% (95% UI: 0.70–6.13) in 1990 to 7.17% (95% UI: 3.30–11.96) in 2019, and was estimated to reach 9.46% (95% UI: 4.40–16.34) by 2040. The extent of increase is roughly the same as that globally (Figure 1A). The total number of deaths attributed to high BMI steadily increased from 0.23 million (95% UI: 0.06–0.51) in 1990 to 0.76 million (95% UI: 0.33–1.31) in 2019; by 2040, this will increase to 1.34 million (95% UI: 0.60–2.44). As shown in Figure 1B, the gap between China and the global increase is gradually widening. However, note that the rate of deaths in China gradually increased during the 30-year monitoring period (19.85 per 100,000 population [95% UI: 4.93–43.41] in 1990 and 53.76 [95% UI: 23.42–92.14] in 2019), and is expected to overtake the world by 2033 and reach 106.23 per 100,000 population (95% UI: 47.23–197.12) by 2040 (Figure 1C), serving as a reminder that overweight and obesity still have serious health consequences.

### 3.2. The Burden of High BMI Is Higher in Men Than in Women

**Figure 2:**
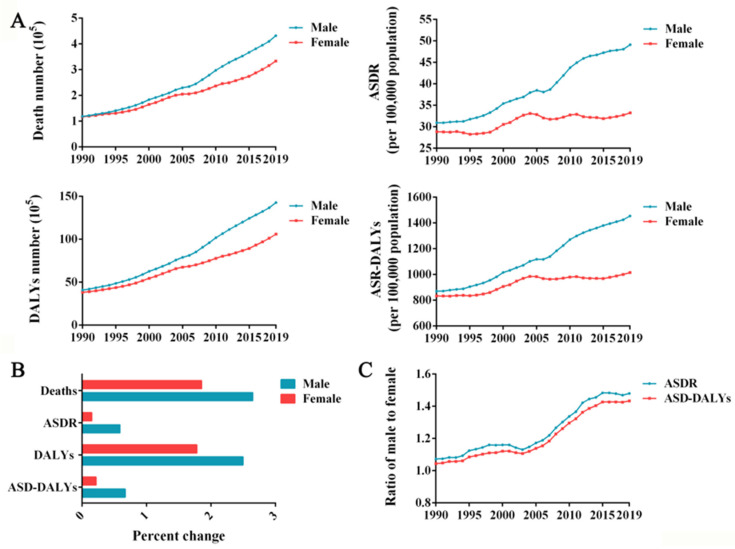
Burden of deaths and DALYs due to high BMI in both sexes in China. (**A**) Deaths, ASDRs, DALYs and ASR-DALYs due to high BMI in men and women in China from 1990 to 2019. (**B**) The percentage changes in deaths, ASDRs, DALYs and ASR-DALYs due to high BMI in men and women between 1990 and 2019. (**C**) Male-to-female ratios of ASDRs and ASR-DALYs due to high BMI in men and women from 1990 to 2019.

The burden of high BMI was higher among men in China than among women, and the sex gap is likely only to widen in the near future. As a result of high BMI, the number of deaths has increased in both men and women over the past decades, reaching 0.43 million (95% UI: 0.19–0.75) deaths in men, and 0.33 million (95% UI: 0.15–0.59) deaths in women in 2019 (Figure 2A). Similarly, DALYs in China were higher for men than for women. The highest DALYs in males (14.25 million (95% UI: 6.51–23.75)) and females (10.58 million (95% UI: 4.98–17.72)) were observed in 2019 (Figure 2A). Over the entire study period, males had higher ASDRs and ASR-DALYs because of high BMI than females, and the gap increased over time (Figure 2A).

Furthermore, the percentage change in deaths, ASDR, DALYs, and ASR-DALYs in females over the study period was less significant than in males (Figure 2B). ASDR ratios of males to females increased from 1.07 in 1990 to 1.48 in 2019, along with ASR-DALY ratios of males to females (Figure 2C). Obviously, high BMI has a greater overall impact on the health of Chinese men than women, and the differences between the sexes in high-BMI-related disease burden has been widening year by year for the past 30 years. The gender gap is likely to widen further if this trend continues. 

### 3.3. High-BMI-related Burden in Adults Exceeds That in Children and Adolescents

**Figure 3:**
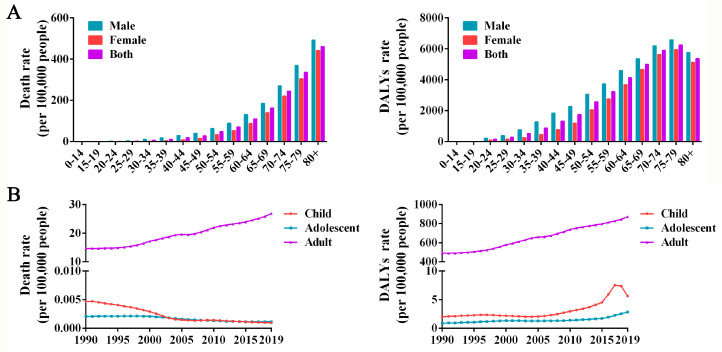
Burden of high BMI in various age groups in China. (**A**) The rate of deaths and DALYs in various age groups in China between 1990 and 2019. (**B**) The rate of deaths and DALYs in adults, children and adolescents between 1990 and 2019 in China.

The burden of high BMI in China is relatively serious, and the rate of deaths and DALYs associated with high BMI increased with age for both men and women (Figure 3A). Men have a higher burden of high BMI than women in all age brackets. At the end of the study period, those over 60 years of age accounted for the majority of deaths and DALYs due to high BMI. After age ≥ 50 years, deaths and DALYs increased rapidly (Figure 3A). As shown in Figure 3B, high-BMI-related disease burden was generally higher among adults (>20 years) than among children (0–14 years) and adolescents (15–19 years). In contrast to high-BMI-related death rates, which continue to increase in adults, it gradually declined from 1990 to 2019 in children and was roughly flat in adolescents. For DALYs rate attributable to high BMI, it increased among children and adolescents after 2015. The peak in the DALYs rate due to high BMI was observed among children in 2017, and declined after that. 

### 3.4. Each Disease Is Affected Differently by a High BMI

**Figure 4:**
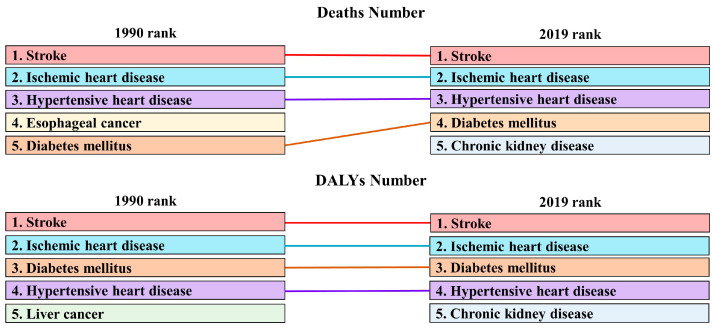
The top 5 high-BMI-related diseases by absolute number of deaths and DLAYs in 1990 and 2019.

In China, chronic non-infectious diseases account for the majority of diseases burden associated with high BMI, including tumors, cardiovascular diseases, diabetes, etc. Over the entire study period, stroke, ischemic heart disease and hypertensive heart disease consistently ranked in the top three for high-BMI-related deaths. The absolute number of deaths caused by chronic kidney disease due to high BMI has increased rapidly, and replaced esophageal cancer as the fifth cause of death associated with high BMI in 2019. Stroke was the leading cause of death related to high BMI in 2019 (266.76 thousand (95% UI: 119.93–453.82)), followed by ischemic heart disease (203.61 thousand (95% UI: 84.29–367.44)) and hypertensive heart disease (73.30 thousand (95% UI: 28.35–142.31)) (Figure 4 and Table 1). In the past 30 years, ASDRs for stroke and ischemic heart disease have increased, but ASDRs for hypertensive heart disease have declined with an EAPC of −0.30 (95% CI: −1.06–0.47).

Moreover, diabetes mellitus replaced hypertensive heart disease as the third largest burden of high-BMI-associated DALYs, and chronic kidney disease cracked the top five for high-BMI-related DALYs, replacing liver cancer. In 2019, stroke was the first cause of high-BMI-related DALYs (8.19 million (95% UI: 3.89–13.43)), followed by ischemic heart disease (5.07 million (95% UI: 2.23–8.74)) and diabetes mellitus (3.73 million (95% UI: 1.91–5.90)) (Figure 4 and Table 2). In addition to liver cancer and asthma, the ASR-DALYs of other high-BMI-related diseases all increased to varying degrees, and the largest increase was observed in kidney cancer, with an EAPC of 5.71 (95% CI: 5.23–6.19). High BMI is also associated with DALYs burden for low back pain, blindness and vision loss, gout and osteoarthritis, but no data confirm that high BMI is responsible for the burden of death from these diseases.

## 4. Discussion

In light of BMI’s high predictive validity, yet unexplained mechanism of action, researchers have proposed an epistemological shift toward viewing BMI as a holistic health assessment rather than as a biomarker [18]. High-BMI-related burden has been globally documented, but has not received enough attention in China [19,20]. In the current work, the temporal trends of death attributed to high BMI in China and globally were assessed over the past 30 years and forecasted for the next 20 years. Our findings show that the burden of death with high BMI annually increased both globally and in China during the observation period. In 2019, a total of 0.76 million deaths were caused by high BMI, and until 2040, this indicator will likely increase to 1.34 million. In 2019, China’s death rate as a result of high BMI has more than doubled since 1990, roughly matching the global trend. As of today, China has one of the highest rates of overweight and obesity in the world [21]. The overweight and obesity epidemic in China will not be reversed if the public does not take preventive measures, and China’s death rate due to high BMI is expected to overtake that of the world in 2033.

Research has shown that both men’s and women’s health can be profoundly affected by gender [22]. We quantified the numbers and ASRs of death and DALYs by sex in China. Both deaths and DALYs caused by high BMI more than doubled between 1990 and 2019. Only a slight increase was observed in women after age-standardizing high-BMI-related deaths and DALYs rates, which was not similar to in men. High-BMI-related deaths and DALYs increased with age for both sexes, and men were more burdened by disease caused by high BMI than women in all age brackets. Gender-based health disparities attributed to high BMI are a result of biological factors and social roles assigned by society [22]. Chinese traditional culture emphasizes that “men should be outside, women should be at home”. Men have more opportunities to go out to socialize than women, which results in men eating more and later, but consuming less than women. Additionally, women’s beauty-loving nature dictates that they pay more attention to weight management than men, and women are willing to take action to maintain a normal range of BMI. The sex differences in high-BMI-related burden have been widening annually over the past 30 years, due to the lack of a sex-based approach to policies and programs developed to target high-BMI-related diseases [23]. The gender gap is likely to widen further if this trend continues. Therefore, the need for ad hoc strategies to address the sex differences with regard to the burden of high-BMI is urgent.

Overall, high-BMI-related burden was generally higher among adults than among children and adolescents. It should be noted that higher children’s BMI is associated with parents’ obesity [24]. Furthermore, compared with high BMI in adults, high BMI in children may also lead to many secondary problems. For example, the caregivers of children with a high BMI missed work 1.3 times a year, while 41% reported 2 or more times as a result of a child’s absence from school [25]. Buscot et al. analyzed BMI trajectories from childhood to adulthood in 2717 young adults, and drew the conclusion that preventing adult obesity should ideally begin before age 6. High BMI naturally resolves in adolescence for males and early adulthood for females, suggesting a critical window for secondary prevention [26]. Children are more likely to have lots of daily activities; therefore, food intake may be an important factor affecting children’s BMI [27]. The good news is that with the popularity of healthy parenting education, younger parents with high levels of education are putting more effort into controlling their children’s weight. This may partly explain the decline in the rate of DALYs with high BMI in children and adolescents after peaking in 2017.

In China, chronic non-infectious diseases account for most of the disease burden associated with high BMI. Over the entire study period, cardiovascular disease consistently ranked in the top three for high-BMI-related deaths. In addition, the leading cause of DALYs associated with high BMI is stroke, followed by ischemic heart disease and diabetes mellitus. It has been recognized that high BMI is often associated with blood lipid disorders, gut bacteria and metabolic disturbances, sterile inflammation, insulin resistance, etc., which together culminate in an increased risk of cardiovascular disease and diabetes mellitus [28,29]. In addition, the burden of kidney cancer and chronic kidney disease related to high BMI are also rising. The poor current and future of high-BMI-related burden in China can be explained as follows: over-eating fish and meat seems to be the consensus of Chinese residents with the rapid growth of the economy; social progress and technological development have offset the huge amount of labor and physical consumption in the past; and unhealthy modern living habits, including irregular eating, eating too fast, sitting for a long time, and staying up late. The obesogenic environment influences people’s eating and physical activity behaviors in complex ways. Lifestyle intervention is the typical way of treating obesity. “Keep your mouth shut and stretch your legs” is the focus of weight loss, which mainly includes adjusting the dietary structure and strengthening exercise [30]. However, for simple obesity with a BMI > 35 or a BMI of 27.5–35, and accompanied by metabolic disease, bariatric surgery may be the only long-term effective method [31].

The potential limitations of our study should also be considered. First of all, the data is not first-hand information, but estimated by GBD2019 based on the global high-BMI-related health burden. Secondly, despite the GBD database’s relatively detailed estimate of China’s high-BMI-related burden, as of now, China assesses the burden of high BMI on a national scale without further analyzing local features. Furthermore, our analysis lacks further stratification of health burden contribute to high BMI according to BMI thresholds due to data loss.

## 5. Conclusions

In China, high BMI represents an urgent problem that must be addressed. A better assessment of changing patterns of high BMI in China may drive public and health policymakers to prioritize this crucial issue. Our findings highlight the need to allocate specific funding, devise strategies and foster behavioral changes to reduce the stress brought on by high BMI. 

## Figures and Tables

**Table 1:** The number of deaths and ASDR in those with high BMI in 1990 and 2019, and their temporal trends from 1990 to 2019.

Characteristics	1990		2019		1990–2019
	Number of Deaths (95% UI)	ASDR per 100,000 (95% UI)	Number of Deaths (95% UI)	ASDR per 100,000 (95% UI)	EAPC of ASDR (95% CI)
Breast cancer	2191.12 (392.14–5324.64)	0.26 (0.05–0.64)	12,058.54 (3393.38–25,189.97)	0.58 (0.16–1.22)	2.94 (2.79–3.09)
Gallbladder and biliary tract cancer	624.53 (142.68–1593.23)	0.08 (0.02–0.21)	3517.53 (1329.62–6901.31)	0.18 (0.07–0.36)	3.53 (3.07–3.98)
Multiple myeloma	100.57 (18.39–261.68)	0.01 (0.00–0.03)	544.26 (169.31–1135.47)	0.03 (0.01–0.06)	2.92 (2.85–3.00)
Leukemia	830.51 (160.30–2103.98)	0.09 (0.02–0.22)	2535.19 (919.85–5039.00)	0.13 (0.05–0.26)	1.54 (1.43–1.65)
Pancreatic cancer	456.04 (78.27–1231.15)	0.06 (0.01–0.15)	4236.32 (1108.12–9601.90)	0.21 (0.06–0.48)	5.02 (4.80–5.24)
Ovarian cancer	71.26 (−1.64–221.51)	0.01 (0.00–0.03)	571.41 (−13.46–1496.10)	0.03 (0.00–0.07)	4.36 (4.22–4.51)
Thyroid cancer	106.51 (22.34–263.82)	0.01 (0.00–0.03)	536.33 (173.56–1120.49)	0.03 (0.01–0.06)	3.08 (2.88–3.27)
Non-Hodgkin lymphoma	225.80 (45.16–598.83)	0.03 (0.01–0.07)	1593.08 (528.29–3315.29)	0.08 (0.03–0.17)	4.58 (4.31–4.86)
Kidney cancer	275.97 (64.58–626.64)	0.03 (0.01–0.08)	2589.07 (1081.71–4699.44)	0.13 (0.05–0.24)	5.61 (5.13–6.09)
Liver cancer	10,362.46 (1928.75–26,875.56)	1.13 (0.21–2.98)	18,964.67 (6165.35–40,278.41)	0.93 (0.30–1.97)	−2.30 (−3.10 to −1.49)
Uterine cancer	1222.16 (315.72–2618.43)	0.14 (0.04–0.30)	2967.44 (1305.70–5508.34)	0.14 (0.06–0.27)	0.36 (−0.26–0.98)
Esophageal cancer	11,782.83 (1921.94–32,264.22)	1.44 (0.23–3.96)	36,180.91 (9426.32–79,605.50)	1.80 (0.47–3.95)	0.64 (0.15–1.13)
Colon and rectum cancer	1817.51 (399.60–4351.24)	0.22 (0.05–0.53)	14,146.85 (5479.98–26,647.74)	0.72 (0.28–1.36)	4.64 (4.35–4.93)
Ischemic heart disease	39,406.17 (9213.83–89,039.24)	5.14 (1.18–12.02)	203,608.68 (84,285.47–367,441.47)	11.20 (4.53–20.83)	3.39 (3.12–3.65)
Hypertensive heart disease	31,034.85 (7085.40–70,850.56)	4.89 (1.00–12.37)	73,299.84 (28,352.47–142,307.78)	4.41 (1.50–9.21)	−0.30 (−1.06–0.47)
Chronic kidney disease	8450.27 (2079.25–18,788.22)	1.08 (0.25–2.52)	37,077.06 (15,764.13–65,371.62)	1.94 (0.80–3.48)	2.46 (2.29–2.64)
Gallbladder and biliary diseases	1845.50 (427.84–4213.17)	0.29 (0.07–0.67)	3052.80 (1234.67–5627.09)	0.19 (0.08–0.35)	−1.81 (−1.95 to −1.67)
Diabetes mellitus	10,510.40 (2937.84–21,513.97)	1.25 (0.34–2.60)	47,529.62 (22,514.23–76,631.88)	2.39 (1.12–3.90)	2.40 (2.10–2.71)
Asthma	2513.82 (548.84–6523.97)	0.39 (0.08–1.04)	3270.05 (1234.85–6226.72)	0.19 (0.07–0.37)	−2.75 (−2.95 to −2.55)
Atrial fibrillation and flutter	975.79 (209.46–2389.03)	0.23 (0.05–0.59)	5876.04 (2152.13–11,856.72)	0.42 (0.15–0.86)	1. 99(1.91–2.06)
Alzheimer’s disease and other dementias	3530.64 (290.14–13,782.39)	0.86 (0.07–3.26)	23,787.11 (3263.72–78,659.44)	1.67 (0.22–5.65)	2.56 (2.46–2.66)
Stroke	106,663.61 (25,825.88–233,294.20)	12.17 (2.91–26.65)	266,755.28 (119,930.67–453,820.00)	13.15 (5.84–22.76)	0.29 (0.07–0.50)

**Table 2:** The number of DALYs and ASR-DALYs in those with high BMI in 1990 and 2019, and their temporal trends from 1990 to 2019.

	1990		2019		1990–2019
Characteristics	Number of DALYs (95% UI)	ASR-DALYs per 100,000 (95% UI)	Number of DALYs (95% UI)	ASR-DALYs per 100,000 (95% UI)	EAPC of ASDR (95% CI)
Breast cancer	62,298.33 (11,393.67–150,486.60)	6.83 (1.24–16.55)	336,582.82 (93,778.62–680,213.61)	15.25 (4.27–30.95)	3.01 (2.83–3.19)
Gallbladder and biliary tract cancer	16,054.25 (3730.29–40,845.03)	1.82 (0.41–4.65)	81,473.34 (31,251.45–157,724.94)	3.95 (1.50–7.65)	3.46 (3.00–3.91)
Multiple myeloma	2828.46 (523.95–7429.59)	0.30 (0.06–0.79)	14,686.71 (4528.44–30,413.52)	0.71 (0.22–1.47)	3.01 (2.94–3.08)
Leukemia	32,518.85 (6123.68–82,756.56)	2.96 (0.57–7.46)	84,302.69 (30,802.27–167,813.73)	4.46 (1.61–8.86)	1.41 (1.29–1.52)
Pancreatic cancer	12,708.94 (2179.22–34,462.91)	1.38 (0.23–3.71)	106,354.05 (27,249.62–240,616.59)	5.10 (1.31–11.54)	4.91 (4.70–5.13)
Ovarian cancer	2352.28 (−52.72–7378.61)	0.24 (−0.01–0.75)	16,701.07 (−398.19–43,257.40)	0.78 (−0.02–2.06)	4.17 (4.01–4.33)
Thyroid cancer	3227.01 (681.69–7943.02)	0.34 (0.07–0.84)	14,811.37 (4838.85–30,476.19)	0.73 (0.24–1.51)	3.07 (2.90–3.23)
Non-Hodgkin lymphoma	7343.51 (1462.21–19,494.52)	0.74 (0.15–1.94)	47,354.30 (16,139.48–99,616.69)	2.34 (0.79–4.91)	4.73 (4.45–5.01)
Kidney cancer	8117.04 (1905.54–18,423.87)	0.86 (0.20–1.95)	70,543.60 (29,312.42–127,228.96)	3.45 (1.44–6.26)	5.71 (5.23–6.19)
Liver cancer	339,787.93 (63,119.03–881,903.59)	34.43 (6.37–89.39)	570,373.28 (183,232.22–1,205,391.20)	27.72 (8.88–58.69)	−2.40 (−3.24 to −1.56)
Uterine cancer	38,377.85 (9763.96–83,099.05)	4.00 (1.02–8.61)	90,828.82 (40,885.43–166,471.87)	4.31 (1.91–7.90)	0.52 (−0.12–1.16)
Esophageal cancer	306,708.88 (49,524.78–827,902.90)	34.17 (5.61–93.35)	859,654.43 (221,497.11–1,875,382.28)	40.79 (10.61–88.68)	0.46 (−0.06–0.98)
Colon and rectum cancer	53,124.79 (11,759.47–127,188.90)	5.68 (1.26–13.62)	376,132.77 (149,260.15–689,707.75)	18.45 (7.30–33.73)	4.69 (4.39–4.98)
Ischemic heart disease	1,183,843. 99(283,867.90–2,643,151.93)	127.54 (30.11–286.07)	5,073,254.14 (2,233,964.46–8,744,122.41)	257.69 (112.34–447.18)	2. 99(2.79–3.19)
Hypertensive heart disease	727,467.32 (176,938.40–1,575,574.30)	91.56 (21.30–208.35)	1,514,532.28 (673,365.89–2,626,320.86)	79.94 (34.45–145.14)	−0.43 (−1.10–0.25)
Chronic kidney disease	297,281.58 (75,327.93–668,658.50)	32.70 (8.15–73.76)	1,200,356.40 (542,874.66–2,014,595.58)	58.48 (26.44–98.96)	2.66 (2.46–2.87)
Gallbladder and biliary diseases	139,950.39 (32,647.51–342,137.02)	15.12 (3.52–36.66)	384,257.75 (158,489.35–746,026.51)	19.26 (7.87–37.13)	0.82 (0.68–0.96)
Diabetes mellitus	771,801.60 (209,707.64–1,609,362.21)	80.21 (21.50–167.40)	3,737,575.96 (1,913,966.15–5,903,925.93)	181.54 (93.71–286.33)	3.11 (2.87–3.34)
Asthma	98,457.09 (25,467.01–228,183.85)	10.89 (2.69–26.17)	175,324.55 (75,051.80–320,742.37)	10.42 (4.61–18.60)	−0.78 (−1.12 to −0.44)
Atrial fibrillation and flutter	40,270.29 (8841.24–97,699.67)	6.03 (1.28–14.78)	228,050.10 (88,026.96–445,218.48)	12.30 (4.71–24.13)	2.57 (2.47–2.67)
Alzheimer’s disease and other dementias	72,968.85 (8730.45–252,707.00)	13.17 (1.56–45.71)	477,208.95 (101,059.42–1,388,765.94)	28.22 (5.68–82.02)	2.81 (2.75–2.88)
Stroke	3,376,373.87 (849,026.71–7,253,492.17)	351.82 (87.09–762.37)	8,188,302.10 (3,891,288.26–13,434,158.41)	397.52 (188.62–654.47)	0.43 (0.26–0.60)
Low back pain	155,486.15 (33,388.62–376,821.46)	15.35 (3.33–36.83)	460,684.90 (184,490.12–899,388.11)	23.37 (9.16–45.50)	2.12 (1.88–2.36)
Blindness and vision loss	9122.33 (1860.75–23,629.60)	1.29 (0.25–3.33)	47,687.87 (15,902.11–101,631.30)	2.50 (0.82–5.35)	2.87 (2.50–3.23)
Gout	18,679.84 (4002.65–47,522.96)	1.94 (0.41–4.97)	115,104.21 (44,197.45–231,849.93)	5.64 (2.16–11.22)	4.28 (4.08–4.49)
Osteoarthritis	99,196.59 (16,850.73–280,351.94)	10.94 (1.84–30.78)	557,902.37 (165,070.11–1,343,750.30)	25. 99(7.67–62.55)	3.40 (3.26–3.54)

## Data Availability

The current study analyzes existing, publicly available data that is available from http://ghdx.healthdata.org/gbdresults-tool (accessed on 31 October 2022). This study did not generate any unique datasets or code. Data made available for download on IHME Websites can be used, shared, modified or built upon by non-commercial users in accordance with the IHME FREE-OF-CHARGE NON-COMMERCIAL USER AGREEMENT.

## References

[1] Caballero B. (2019). Humans against Obesity: Who Will Win?. Adv. Nutr..

[2] Dikaiou P., Björck L., Adiels M., Lundberg C.E., Mandalenakis Z., Manhem K., Rosengren A. (2021). Obesity, overweight and risk for cardiovascular disease and mortality in young women. Eur. J. Prev. Cardiol..

[3] Yang Y., Shields G.S., Guo C., Liu Y. (2018). Executive function performance in obesity and overweight individuals: A meta-analysis and review. Neurosci. Biobehav. Rev..

[4] Yu K.M., Liu X., Alhamzawi R., Becker F., Lord J. (2018). Statistical methods for body mass index: A selective review. Stat. Methods Med. Res..

[5] Bode E.D., Mathias K.C., Stewart D.F., Moffatt S.M., Jack K., Smith D.L. (2021). Cardiovascular Disease Risk Factors by BMI and Age in United States Firefighters. Obesity.

[6] Wei J., Liu X., Xue H., Wang Y., Shi Z. (2019). Comparisons of Visceral Adiposity Index, Body Shape Index, Body Mass Index and Waist Circumference and Their Associations with Diabetes Mellitus in Adults. Nutrients.

[7] Azhar A., Hassan N., Tapolyai M., Molnar M.Z. (2021). Obesity, Chronic Kidney Disease, and Kidney Transplantation: An Evolving Relationship. Semin. Nephrol..

[8] Xing J., Guan X., Zhang Q., Chen S., Wu S., Sun X. (2021). Triglycerides Mediate Body Mass Index and Nonalcoholic Fatty Liver Disease: A Population-Based Study. Obes. Facts.

[9] Zhang G., Cao F., Shi L., Ma T., Zhang L. (2020). Contribution of high body mass index and alcohol use to liver cancer-related mortality: A study based on 195 countries or territories. Dig. Liver Dis..

[10] Kim J., So W.Y. (2018). High Body Mass Index is Associated with the Extent of Muscle Damage after Eccentric Exercise. Int. J. Environ. Res. Public Health.

[11] Titchener K., Wong Q.J. (2015). A weighty issue: Explaining the association between body mass index and appearance-based social anxiety. Eat. Behav..

[12] Horenstein A., Kaplan S.C., Butler R.M., Heimberg R.G. (2021). Social anxiety moderates the relationship between body mass index and motivation to avoid exercise. Body Image.

[13] Afshin A., Forouzanfar M.H., Reitsma M.B., Sur P., Estep K., Lee A., Marczak L., Mokdad A.H., Moradi-Lakeh M., GBD 2015 Obesity Collaborators (2017). Health Effects of Overweight and Obesity in 195 Countries over 25 Years. N. Engl. J. Med..

[14] Dai H., Alsalhe T.A., Chalghaf N., Riccò M., Bragazzi N.L., Wu J. (2020). The global burden of disease attributable to high body mass index in 195 countries and territories, 1990-2017: An analysis of the Global Burden of Disease Study. PLoS Med..

[15] Sung H., Siegel R.L., Torre L.A., Pearson-Stuttard J., Islami F., Fedewa S.A., Goding Sauer A., Shuval K., Gapstur S.M., Jacobs E.J. (2019). Global patterns in excess body weight and the associated cancer burden. CA Cancer J. Clin..

[16] Pandey A.R., Chalise B., Shrestha N., Ojha B., Maskey J., Sharma D., Godwin P., Aryal K.K. (2020). Mortality and risk factors of disease in Nepal: Trend and projections from 1990 to 2040. PLoS ONE.

[17] Wang W.X., Hu K.L., Liu H., Zhang X.Y., Li H.M., Zhou F., Liu Y., Lei F., Qin J., Zhao Y. (2021). Global Burden of Disease Study 2019 suggests that metabolic risk factors are the leading drivers of the burden of ischemic heart disease. Cell. Metab..

[18] Gutin I. (2018). In BMI We Trust: Reframing the Body Mass Index as a Measure of Health. Soc. Theory Health.

[19] Felisbino-Mendes M.S., Cousin E., Malta D.C., Machado I.E., Ribeiro A.L.P., Duncan B.B., Schmidt M.I., Silva D.A.S., Glenn S., Afshin A. (2020). The burden of non-communicable diseases attributable to high BMI in Brazil, 1990–2017: Findings from the Global Burden of Disease Study. Popul. Health Metr..

[20] Lin X., Xu Y., Xu J., Pan X., Song X., Shan L., Zhao Y., Shan P.F. (2020). Global burden of noncommunicable disease attributable to high body mass index in 195 countries and territories, 1990–2017. Endocrine.

[21] Wang Y., Wang L., Qu W. (2017). New national data show alarming increase in obesity and noncommunicable chronic diseases in China. Eur. J. Clin. Nutr..

[22] Gahagan J., Gray K., Whynacht A. (2015). Sex and gender matter in health research: Addressing health inequities in health research reporting. Int. J Equity Health.

[23] Alexander S., Hayes S. (2017). Viewing Health Policy through a Gender Lens: Highlights from Several U.S. Communities. Womens Health Issues.

[24] Brand C., Fochesatto C.F., Dias A.F., Gaya A.R., Martins C.M.L., Renner J.D.P., Reuter C.P., Kelishadi R. (2021). Child’s body mass index and mother’s obesity: The moderating role of physical fitness. Eur. J. Pediatr..

[25] Lee J., Kubik M.Y., Fulkerson J.A. (2021). Missed Work Among Caregivers of Children with a High Body Mass Index: Child, Parent, and Household Characteristics. J. Sch. Nurs..

[26] Buscot M., Thomson R.J., Juonala M., Sabin M.A., Burgner D.P., Lehtimäki T., Hutri-Kähönen N., Viikari J.S.A., Jokinen E., Tossavainen P. (2018). BMI Trajectories Associated with Resolution of Elevated Youth BMI and Incident Adult Obesity. Pediatrics.

[27] Huang J.Y., Qi S.J. (2015). Childhood obesity and food intake. World J. Pediatr..

[28] Andersen C.J., Murphy K.E., Fernandez M.L. (2016). Impact of Obesity and Metabolic Syndrome on Immunity. Adv. Nutr..

[29] Gomes A.C., Hoffmann C., Mota J.F. (2018). The human gut microbiota: Metabolism and perspective in obesity. Gut Microbes.

[30] Mozaffarian D. (2016). Dietary and Policy Priorities for Cardiovascular Disease, Diabetes, and Obesity: A Comprehensive Review. Circulation.

[31] Piche M.E., Tchernof A., Despres J.P. (2020). Obesity Phenotypes, Diabetes, and Cardiovascular Diseases. Circ. Res..

